# Impact of Westernized Diet on Gut Microbiota in Children on Leyte Island

**DOI:** 10.3389/fmicb.2017.00197

**Published:** 2017-02-14

**Authors:** Jiro Nakayama, Azusa Yamamoto, Ladie A. Palermo-Conde, Kanako Higashi, Kenji Sonomoto, Julie Tan, Yuan-Kun Lee

**Affiliations:** ^1^Laboratory of Microbial Technology, Department of Bioscience and Biotechnology, Faculty of Agriculture, Kyushu UniversityFukuoka, Japan; ^2^PhilRootcrops, Visayas State UniversityBaybay, Philippines; ^3^Department of Microbiology, National University of SingaporeSingapore, Singapore

**Keywords:** gut microbiota, 16S rRNA gene sequencing, Firmicutes, Bacteroidetes, *Prevotella*, high-fat diet, obesity, Philippines

## Abstract

Urbanization has changed life styles of the children in some towns and cities on Leyte island in the Philippines. To evaluate the impact of modernization in dietary habits on gut microbiota, we compared fecal microbiota of 7 to 9-year-old children from rural Baybay city (*n* = 24) and urban Ormoc city (*n* = 19), and assessed the correlation between bacterial composition and diet. A dietary survey indicated that Ormoc children consumed fast food frequently and more meat and confectionary than Baybay children, suggesting modernization/westernization of dietary habits. Fat intake accounted for 27.2% of the total energy intake in Ormoc children; this was remarkably higher than in their Baybay counterparts (18.1%) and close to the upper limit (30%) recommended by the World Health Organization. Their fecal microbiota were analyzed by high-throughput 16S rRNA gene sequencing in conjunction with a dataset from five other Asian countries. Their microbiota were classified into two enterotype-like clusters with the other countries’ children, each defined by high abundance of either Prevotellaceae (P-type) or Bacteroidaceae (BB-type), respectively. Baybay and Ormoc children mainly harbored P-type and BB-type, respectively. Redundancy analysis showed that P-type favored carbohydrates whereas BB-type preferred fats. Fat intake correlated positively with the Firmicutes-to-Bacteroidetes (F/B) ratio and negatively with the relative abundance of the family Prevotellaceae/genus *Prevotella*. A species-level analysis suggested that dietary fat positively correlated with an *Oscillibacter* species as well as a series of *Bacteroides/Parabacteroides* species, whereas dietary carbohydrate positively correlated with *Dialister succinatiphilus* known as succinate-utilizing bacteria and some succinate-producing species of family *Prevotellaceae, Veillonellaceae*, and *Erysipelotrichaceae.* We also found that a *Succinivibrio* species was overrepresented in the P-type community, suggesting the syntroph via hydrogen and succinate. Predicted metagenomics suggests that BB-type microbiota is well nourished and metabolically more active with simple sugars, amino acids, and lipids, while P-type community is more involved in digestion of complex carbohydrates. Overweight and obese children living in Ormoc, who consumed a high-fat diet, harbored microbiota with higher F/B ratio and low abundance of *Prevotella*. The altered gut microbiota may be a sign of a modern diet-associated obesity among children in developing areas.

## Introduction

Globalization has affected people’s eating habits, leading many of them to consume high-fat and high-calorie foods (Shridhar et al., 2015). Modern diets have adverse effects on human health and raise global issues, particularly for young generation in developing areas (Imamura et al., 2015). Diets shape the gut microbial community by supplying the nutrients and conditioning the intestinal microenvironment (De Filippo et al., 2010; Wu et al., 2011; Lee, 2013; Zhang et al., 2014; De Filippis et al., 2015). Gut microbes interact with the host through cell components, metabolites, and enzymes, some of which play a crucial role in promotion or maintenance of host’s health (Fukuda et al., 2011; Kinross et al., 2011; Furusawa et al., 2014; Donia and Fischbach, 2015; Hevia et al., 2015). Specifically, bacterial colonization is required for the ordinary development, maturation, and maintenance of the host immune system; whereas perturbation of gut microbial community can induce an inflammatory state that causes metabolic disorder leading to obesity and various diseases (Sudo et al., 1997; Carvalho et al., 2012; Zhao, 2013; Cavalcante-Silva et al., 2015).

The link between diet and gut microbiota has been studied extensively (De Filippo et al., 2010; Wu et al., 2011; Zhang et al., 2014; De Filippis et al., 2015; O’Keefe et al., 2015). It ranges from short- to long-term dietary effects and from local to global structural changes in the gut microbial community. Ley et al. (2006) found that low-calorie diets in obese subjects efficiently altered the abnormal balance of the two dominant phyla, Firmicutes and Bacteroidetes, so-called F/B ratio, to a level comparable with that of normal-weight individuals. On the other hand, Duncan et al. (2008) reported that weight-loss diets changed the composition of microbiota at a species rather than a phylum level. Comparable results have been obtained in some following studies in mice or humans, although the results have not been consistent and the relationship between F/B ratio and obesity have been controversial (Backhed et al., 2005; Duncan et al., 2008; Turnbaugh et al., 2009; Murphy et al., 2010; Schwiertz et al., 2010; Bell, 2015; Kasai et al., 2015; Remely et al., 2015; Louis et al., 2016).

The effect of long-term diets has been investigated in epidemiological studies by comparing gut microbial composition between populations with different dietary habits. De Filippo et al. (2010) reported a considerable difference in gut microbiota at a phylum level, namely F/B ratio, between African children who followed a low-fat high-fiber diet, and Italian children who consumed a high-fat and high-protein modern diet. In addition to these phylum-level community variations, “enterotypes” represent global type varieties of human gut microbial community structures, that are observed over the world (Arumugam et al., 2011; Koren et al., 2013; Nakayama et al., 2015). Although enterotype clustering is controversial because it depends on the clustering model used and boundaries between clusters become less clear-cut with increase of sample sizes (Jeffery et al., 2012; Koren et al., 2013; Knights et al., 2014; Gorvitovskaia et al., 2016), several studies have indicated the existence of at least two types of microbiomes comprising a trade-off of *Prevotella* or *Bacteroides* within or across cohort, or further within individual over time (Wu et al., 2011; Ou et al., 2013; Ding and Schloss, 2014; Lim et al., 2014; Zhang et al., 2014, 2015; Nakayama et al., 2015; Trosvik and de Muinck, 2015; Vandeputte et al., 2015; Oki et al., 2016). The enterotypes are reportedly associated with high-fat high-protein, and low-fat high-carbohydrate diets (Wu et al., 2011; Zhang et al., 2014). The former correspond to modern artificial foods with a high content of animal fat and protein. The latter include traditional low-fat diets with a high content of plant polysaccharides or meat-free vegetarian diets (Glick-Bauer and Yeh, 2014; Ruengsomwong et al., 2016).

The Asian Microbiome Project (AMP) aims to investigate the link between different traditional diets, gut microbiota, and Asians’ health. Phase I focused on school-age children, whose dietary habits essentially reflected those of their country. As a result of 16S rRNA amplicon sequence analysis of 303 subjects from five countries, namely, China, Japan, Taiwan, Thailand, and Indonesia, we have identified two enterotype-like variations, each characterized by high abundance of *Prevotella*, P-type, and *Bacteroides*/*Bifidobacterium*, BB-type (Nakayama et al., 2015). Preponderance of either enterotype was observed in each country, suggesting the link of their staple foods with enterotype. However, even within a country, the gut microbiota sometimes differed significantly between regions, notably in developing area having a profound influence by modernization/westernization. Thus, we suspect the impact of change in dietary habit on host gut microbiota and conduct Phase III study to focus on specific populations with distinct dietary habits.

**FIGURE 1:**
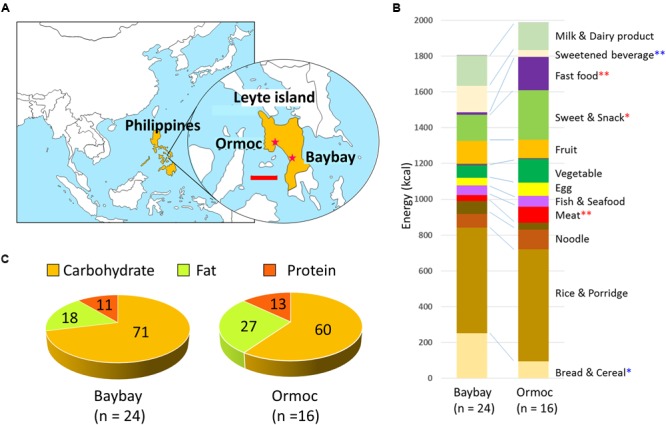
**Geographic location of Baybay and Ormoc, and dietary habits of school-age children in these cities. (A)** Map of east and southeast Asia with a detailed view of Leyte island indicating the location of Baybay and Ormoc. The scale bar in the enlarged map indicates 50 km. **(B)** Composition of daily dietary intake in children of Baybay (*n* = 24) and Ormoc (*n* = 16). The contribution of each food was estimated from the parents’ answers to the food frequency questionnaire (FFQ) and was converted to energy units (kcal) according to the databases of energy and nutrition composition of food. The average per city is presented. Red and blue asterisks indicate significantly higher in Baybay and Ormoc children, respectively, with ^∗^*p* < 0.05 (single asterisk) or ^∗∗^*p* < 0.001 (double asterisks) in Wilcoxon rank-sum test. Variation across individual of this dataset is shown in Supplementary Figure S2. **(C)** Nutrient composition (in %) of the overall dietary intake in children from Baybay and Ormoc. The energy ratio of macronutrients (kcal/day) was estimated according to the same data and databases as in **(B)**, averaged per city, and represented as pie charts. Variation across individual of this dataset is shown in Supplementary Figure S3.

To begin the AMP Phase III study, we investigate on preadolescent children in two cities on Leyte island. Leyte is one of the larger islands in the Philippines, located in the Eastern Visayas region (**Figure 1A**). Ormoc is the second most populous city on Leyte. According to the Philippines six-stage income classification of cities, Ormoc is a first class city, whereas Baybay corresponds to a fourth class city. Ormoc has a mixture of modern and traditional features; Baybay is a typical rural town in the Philippines, where residents maintain traditional dietary habits. In this study, we compared fecal bacterial composition and dietary records of school-age children from these two cities. Most notably, we observed a shift in the enterotype-like structure between Ormoc and Baybay children, as indicated by the effect of a high-fat diet. It attracts interest to study such a dynamic shift in gut microbiota between two populations living on only 50 km apart on the same island, as have observed across the continents in previous studies (De Filippo et al., 2010; Yatsunenko et al., 2012). Our results suggest a considerable impact of modern diets on the gut microbiota of children in developing areas.

## Materials and Methods

### Study Design

We recruited 43 children, aged 7 to 9, who were born and raised in Baybay city (*n* = 24) or Ormoc city (*n* = 19). The parents/guardians answered a questionnaire that addressed the children’s physiological characteristics and ongoing health conditions including antibiotic administration record within 2 weeks prior to sampling. Two subjects coded as No.2 and No.11 administered amoxicillin 2 days and 1 week before sampling, respectively. Except for three subjects of mix blood of Filipino with Japanese, Tongan, or Malay, all subjects were pure Filipinos. Characteristics of the participants in this study was summarized in **Table 1**. There was no statistical difference for gender (male/female ratio) or age between Baybay and Ormoc children, whereas height, weight, and body mass index (BMI) statistically differed between these two groups. This study was approved by the ethics committees of the Faculty of Agriculture of Kyushu University, and the NUS Institutional Review Board. Written informed consent was obtained from the parents/guardians of all participants. We entered and analyzed all samples and questionnaire data anonymously and published them using identification numbers of the participants.

**Table 1:** Characteristics of the participants in this study.

	Baybay (*n* = 24)	Ormoc (*n* = 19)	*p*^∗^
Age (y)	8.21 @ 0.51	8.11 @ 0.66	0.62
Gender (male/female)	14/10	12/7	0.77
Weight (kg)	21.3 @ 2.7	31.1 @ 8.2	<0.05
Height (cm)	119.8 @ 4.0	129.1 @ 8.6	<0.05
BMI (kg/m^2^)	14.8 @ 1.4	18.8 @ 4.5	<0.05

*^∗^Except for gender, the statistical significance was assessed by the Wilcoxon rank sum test. For gender, the statistical significance was assessed by the Fisher’s exact test.*

### Dietary Information

The eating habits (over the previous 3 months) of participating children were surveyed by interview to their parents/guardians using a food frequency questionnaire (FFQ), that included 137 food and drink items. The FFQ was modified from the Singapore National Dietary Survey (Report of the National Nutrition Survey, 2010, Health Promotion Board, Singapore) and adapted to the dietary habits of Filipino children. The nutrient composition and energy level of each food item in the survey was estimated based on the databases of energy and nutrition composition of foods prepared by the Ministry of Health of Singapore or by Food and Nutrition Research Institute, Philippines. The daily intake of nutrients and energy was summarized and compared to Japanese children at similar age (Supplementary Table S1). Total daily intake of each nutrient was summed up for each individual. The total dietary energy intake of each participant was subjected to ‘grubbs test’ (Grubbs, 1950) in the R outliers package to detect outliers. As a result, we established 2907.5 kcal/day as the highest threshold; this led to the exclusion of the nutrient records of three participants (No. 35, 40, and 45, see Supplementary Figure S1 for details of distribution of samples and outliers). These three subjects were excluded for the analysis related to the intake of nutrients, whereas they were retained for the other analyses. The three macronutrients (protein, fat, and carbohydrate), whose intake and energy contribution values were normally distributed (*p* > 0.05 in Shapiro–Wilk and *F*-tests), were statistically compared between Ormoc and Baybay children by Student’s *t*-test. A *post hoc* power analysis was conducted with G^∗^Power software 3.1.9.2 (Faul et al., 2007) to retrospectively examine the observed power in these tests. The differences in the other nutrient intake were examined statistically using the Wilcoxon rank-sum test in Stata SE12.0 (Stata Corporation, College Station, TX, USA).

### Stool Sample Collection and Processing

Stool sampling and processing were performed according to previously described methods established in the AMP Phase I study (Nakayama et al., 2015). Fresh feces were collected in separate sterile feces tubes (Sarstedt, Nümbrecht, Germany) containing 2 mL of RNAlater (Ambion, Inc., Austin, TX, USA). The samples were transferred to the laboratory within 15 h and then stored at -20°C. DNA was extracted from stool samples using the bead-beating method as previously described (Matsuki et al., 2004).

### High-Throughput 16S rRNA Gene Sequencing and Data Processing

The high-throughput 16S rRNA gene sequence analysis was conducted as described in our previous work (Nakayama et al., 2015). The V6–V8 region of the bacterial 16S rRNA gene was amplified by 20 cycles PCR using the barcode-tag universal primer sets Q-968F-# and Q-1390R-# (where # indicates a series of barcode sequence tags) (Nakayama, 2010). The amplicons of the 43 samples were mixed into one batch and subjected to pyrosequencing using a Roche 454 Genome Sequencer with FLX titanium system (Roche Diagnostics, Basel, Switzerland). The raw sequence data were deposited at the DNA Data Bank of Japan (DDBJ) sequence read archive (DRA005273) under BioProject no. PRJDB1664, which contains links and access to stool sampling data under BioSample SAMD00066807 to SAMD00066849.

The sequence data were processed using the Quantitative Insights Into Microbial Ecology (QIIME) pipeline software version 1.8.0^1^ (Caporaso et al., 2010) as described previously (Nakayama et al., 2015). First, batch sequences were sorted into each sample set by applying the function split_libraries.py to barcode sequences, while these were subjected to quality filtering using minimum average quality score of 25. The resulting set of de-multiplexed sequences (600,000 sequences) was combined with the AMP Phase I dataset (DRA001863 to DRA001872) based on 303 children from China, Japan, Taiwan, Thailand, and Indonesia. A total of 2,304,482 sequences were subjected to USEARCH v.5.2.236 to construct operational taxonomic units (OTUs) consisting of sequences with more than 97% identity and to remove PCR chimeras (Edgar, 2010; Edgar et al., 2011) and OTUs consisting of only one read (singletons). As a result, 6,740 OTUs with quality-filtered sequences were obtained. The read count of each OTU in the 346 samples was tabulated as an OTU table using the make_otu_table.py command in the QIIME pipeline. Eight samples from Phase I with fewer than 2,000 read counts were excluded and eventually data for 338 samples containing all 43 Filipino samples were obtained with 6,544 ± 3,401 sequences per sample.

Representative sequences of each OTU were selected with the pick_rep_set.py command. The taxonomy of these representative sequences was analyzed by applying the ‘uclust’ consensus-taxonomy assignment (assign_taxonomy.py) to the Greengenes reference sequence database (gg_13_5) (DeSantis et al., 2006). Bacterial composition of each fecal sample was determined for each taxonomic rank by applying the summarize_taxa_through_plots.py function to the OTU table with the assigned taxonomy dataset. For species, the OTU table was subsampled with ten random iterations to adjust the sampling depth to 2,000 reads, by the multiple_rarefactions.py program in the QIIME pipeline, and then applied to the summarize_taxa_through_plots.py program at level 7. For unclassified taxonomic groups, we searched for closest species by using RDP Sequence Match search (Cole et al., 2005) in the Ribosomal Database Project II^2^ with the sequence of corresponding OTU. The cut-off value (S_ab score) in the Sequence Match search was set at 0.84. If more than two species showed the same highest score, the one with the highest count among the top 20 matches was selected for annotating the species by using a Microsoft Excel macro file named Seqmatch Q400 (Nakayama, 2010).

Alpha rarefaction was performed by using the QIIME alpha_rarefaction.py script on the OTU table. Alpha diversity metrics of observed_species (the number of observed OTUs), PD_whole_tree (Kuczynski et al., 2012) and Shannon–Weiner (Shannon, 1948) were calculated with 10 random iterations at varying sampling depth.

### Principal Component Analysis (PCA) and Clustering Analysis

To perform PCA of 338 samples, their family level bacterial compositions were subjected to the ‘prcomp’ function in R 3.0.2. Subsequently, clustering was performed according to the enterotyping tutorial in R (EMBL^3^) as previously described (Arumugam et al., 2011; Nakayama et al., 2015). Briefly, Jensen–Shannon divergence (Endres and Schindelin, 2003) of 338 samples was calculated based on the same dataset used for PCA. Partitioning around medoids clustering was performed based on the distance matrix. The optimal number of clusters was chosen by maximizing the Calinski–Harabasz index (‘index.G1’ function in the R library ‘clusterSim’)^4^ (Calinski and Harabasz, 1974), and the obtained cluster was validated by prediction strength (Tibshirani and Walther, 2005) and silhouette index (Rousseeuw, 1987). The result of clustering was visualized with a PCA plot using ‘s.class’ function in the R ade4 package^5^ (Dray and Dufour, 2007).

### Correlation Analysis between Dietary Nutrients and Fecal Bacterial Community

Distance-based redundancy analysis (db-RDA) (Legendre and Anderson, 1999) was performed to correlate macronutrient intake with fecal bacterial composition. The individual datasets (*n* = 24 for Baybay; *n* = 16 for Ormoc) of macronutrient energy contributions and family level bacterial composition were subjected to the ‘capscale’ function in the R vegan package^6^. The Bray–Curtis distance was used to coordinate bacterial community variation. The covariable effect between dietary nutrients and city of residence was estimated by the conditioning function in the ‘capscale’ function. Values of *R*^2^ and *p* were estimated by the ‘RsquareAdj’ and ‘Anova’ functions in the R vegan package, respectively.

We also analyzed the correlation between each dietary nutrient and fecal bacterial community using the ‘envfit’ function in the R vegan package (Oksanen, 2015). The correlation was calculated using individual macro- and micronutrient intakes and family level PCA data. The correlation between fat intake and the relative abundance of each bacterial taxonomic group was calculated in Stata SE12.0 by Spearman’s rank correlation test, using data on the fat energy ratio and relative abundance of each taxonomic group.

To create an association network map among bacteria species and three macronutrients, we calculated pairwise Spearman’s correlation coefficients between species and between species and each macronutrient, according to the relative abundance of species and energy ratio of three macronutrient of Leyte children. The Spearman’s correlation coefficients (rho) were obtained with *p-*value by using the out.association program in Mothur software ver. 1.35.1^7^ (Schloss et al., 2009). Correlation coefficients higher than 0,4 or lower than -0.4 with *p* < 0.05 were extracted and visualized as edges connecting two species nodes in the Prefuse Force Directed Layout using Cytoscape 3.3.0^8^ (Lopes et al., 2010).

### PICRUSt (Phylogenetic Investigation of Communities by Reconstruction of Unobserved States)

Phylogenetic investigation of communities by reconstruction of unobserved states analysis (Langille et al., 2013) was performed by using the online galaxy version^9^. 97% OTUs are picked using a closed-reference OTU picking protocol (QIIME 1.8.0) against the Greengenes database pre-clustered at 97% identify (GG 13.5). The obtained OTU table was normalized by 16S rRNA copy number. Then, the number of each KEGG gene in each sample was counted using a KEGG gene content table (ko_13_5). Finally, the abundances of KEGG pathways in each sample were estimated at each KEGG hierarchy level using KEGG catalog^10^ (Kanehisa and Goto, 2000).

### Correlation between Fecal Microbiota and Host Physiology

Due to the lack of record for height, No.37 from Ormoc was excluded from the correlation analysis between fecal microbiota and host physiology. According to the recommended classification of overweight and obese children, the 85 and 95th percentiles were used for the classification into overweight and obesity groups, respectively. Subjects below 15th percentile were classified as underweight. Individual fat energy intake level, which is normally distributed among Leyte children, was statistically compared between the overweight-obese group and normal-underweight group by the Student’s *t*-test in Stata SE12.0. Statistical difference in the F/B ratio and *Prevotella* abundance between these two groups were examined by the Wilcoxon rank-sum test in Stata SE12.0. A *post hoc* power analysis was conducted with G^∗^Power software 3.1.9.2 to retrospectively examine the observed power in these tests.

## Results

### Difference in Dietary Habits between Baybay and Ormoc Children

Diets differed significantly between Baybay and Ormoc children (**Figure 1B**, for details, see Supplementary Figure S2). In particular, 93.8% of Ormoc children ate fast food in the past 3 months at a frequency of 4.0 times a week on average, whereas 41.7% of Baybay children did so at a frequency of 0.9 times a week on average. Also, Ormoc children consume considerable amounts of confectionary such as biscuit and sweetened pastry (276 kcal/d) that corresponded to about double amount eaten by Baybay children (147 kcal/d). Further, meat consumption significantly differed between these two cities (33 ± 23 kcal/d in Baybay and 91 ± 59 kcal/d in Ormoc), although the level was still lower than those consumed by the children in developed countries. These reflect the modernization/westernization of dietary habits in Ormoc children.

The energy ratio of consumed macronutrients (**Figure 1C**) showed that the Ormoc children consumed relatively high-fat, high-protein, and low-carbohydrate diet compared to Baybay children. Particularly, Ormoc children consumed significantly more fat than Baybay children (*p* < 0.001 in Student’s *t*-test, Supplementary Figure S3). The *post hoc* power analysis indicated the power more than 0.8 for the energy ratio of the three macronutrients (Supplementary Figure S3), suggesting that the sample size was enough to detect significant difference in the dietary habit among Leyte children. The total fat intake of Ormoc children was on average 60.1 g/day (27.2% per total energy intake), whereas that of Baybay children was 36.3 g/day (18.1%). As a reference, fat intake in Japanese children (7 to 14-year-old) was 63.8 g/day (28.9%; Ministry of Health, Labour, and Welfare, Japan, 2014) and that of American children (6 to 11-year-old) was 71.1 g/day (33.0%) (Enns et al., 2002). Therefore, Ormoc children consumed fat at a level comparable to that of Japanese and slightly below that of American children. It should be noted that the World Health Organization recommends overall fat intake not to exceed 30% of total energy intake (Hooper et al., 2012). Accordingly, three subjects from Ormoc were identified as having consumed fats above the recommended range.

### Characteristics of the Gut Bacterial Community in Leyte Children

**FIGURE 2:**
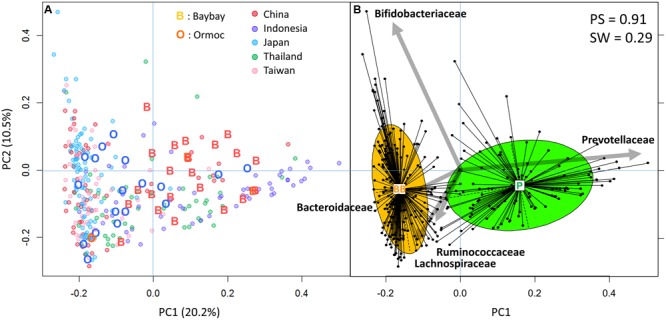
**Principal component analysis (PCA) and clustering of gut bacterial community of 43 Leyte children and 295 Asian Microbiome Project (AMP) Phase I children. (A)** PCA plot of 338 children samples. Family level bacterial composition of 338 samples was subjected to PCA and the first two principal components, PC1 and PC2, were plotted. “O,” Ormoc children; “B,” Baybay children; colored circles, countries of the AMP Phase I study. Percentage values in parentheses next to PC1 and PC2 represents percentage of variance explained by each component. **(B)** Clustering of the 338 samples. The family level composition data were subjected to Jensen–Shannon divergence and partitioning around medoids cluster analysis. To maximize the Calinski–Harabasz index, an optimal number of clusters was chosen; the result was then validated based on prediction strength (PS) and average silhouette width (SW). The clustering result is displayed using the PCA plot. The center of gravity of each cluster is indicated by a rectangle with the name of a type of microbiota. The colored ellipse covers 67% of the samples belonging to a cluster. The five largest PCA loadings of bacterial families are indicated by arrows next to their family names.

We profiled the fecal bacterial composition of 43 children by means of high-throughput 16S rRNA gene sequence analysis. The results were compared with those of the AMP Phase I dataset containing samples from 295 children from five countries (Nakayama et al., 2015). We used their family level bacterial composition to perform PCA (**Figure 2A**) followed by clustering analysis (**Figure 2B**). As observed also in the Phase I study, we obtained two clusters with weak but significant cross-validation scores (**Figure 2B**). The right-side green cluster was characterized by Prevotellaceae and the left-side orange cluster was characterized by the other four dominant families: Bacteroidaceae, Bifidobacteriaceae, Ruminococcaceae, and Lachnospiraceae. According to the definition of the Phase I study, these types of microbiota were identified as P-type and BB-type, respectively. Even after adding the Filipino samples, all 295 Phase I samples, except two, could be assigned to the same type as in the Phase I study, indicating robustness of microbiota typing. The P-type cluster contained more Indonesian and Thai samples, while the BB-type cluster encompassed mostly Japanese, Taiwanese, and Chinese samples. Filipino samples were distributed into both clusters, however, Baybay samples were included mainly in the P-type cluster (87.5%), and Ormoc samples in the BB-type (78.9%). The different preponderance of microbiota types in the two cities on Leyte island suggests the existence of a regional factor affecting gut microbial composition.

**FIGURE 3:**
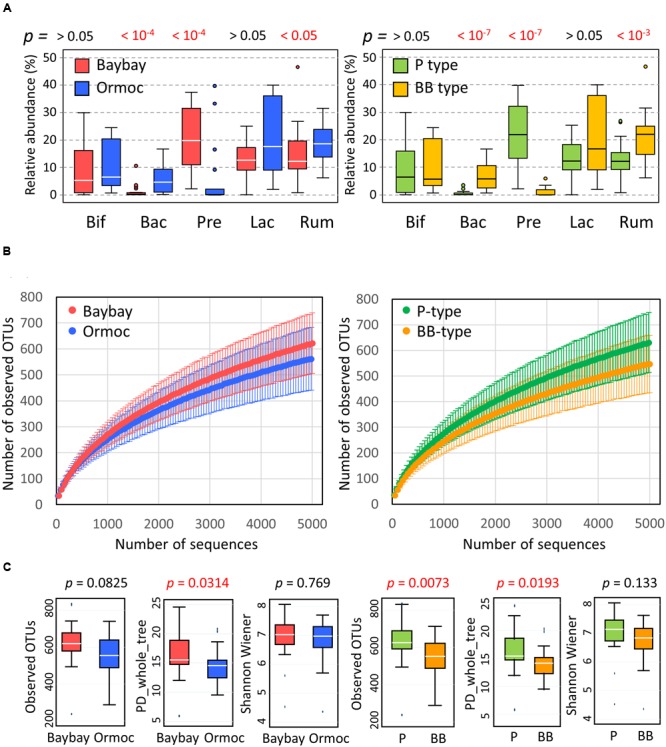
**Characteristics of the gut bacterial community in Leyte children. (A)** Relative abundance of five major bacterial families in fecal samples of Baybay (*n* = 24) and Ormoc (*n* = 19) children (left panel) and of P-type (*n* = 25) and BB-type (*n* = 18) children (right panel). The box plots show the smallest and largest values, 25 and 75% quartiles, the median, and outliers. Bif, Bifidobacteriaceae; Bac, Bacteroidaceae; Pre, Prevotellaceae; Lac, Lachnospiraceae, Rum, Ruminococcaceae. The Wilcoxon rank-sum test was performed to detect statistical differences between the two cities and the *p*-value is shown over the tested pair of box plots. **(B)** Rarefaction curve of the number of OTUs observed in samples from Baybay and Ormoc children (left panel) and from P-type and BB-type children (right panel). The number of OTUs was determined in each sample at each sequencing depth. The mean and standard deviation of each group are shown in the rarefaction plot. **(C)** Alpha-diversities of fecal bacterial community in individual samples from Baybay and Ormoc children (left three panels) and from P-type and BB-type children (right three panels). Individual OTU-composition data (OTU table) were rarified using 5,000 reads per participant in ten iterations. The number of observed OTUs, PD_whole_tree, and Shannon–Wiener index were calculated for each rarified OTU composition and averaged within the 10 iterations. The covariance of these calculated indices was computed for each country and was graphed as a box plot showing the smallest and largest values, 25 and 75% quartiles, the median, and outliers. It is noted that Shannon–Wiener index represents an entropic index but not itself diversity which should be expressed as the exponential of this value.

We compared the relative abundance of the above five families of bacteria between two cities (**Figure 3A**, left panel). Prevotellaceae were clearly more abundant in Baybay than in Ormoc children, whereas Bacteroidaceae showed the opposite distribution. In particular, Prevotellaceae accounted for more than 10% of the total community in the majority of Baybay children, while representing less than 1% in the majority of Ormoc children. Prevotellaceae and Bacteroidaceae included only the genera *Prevotella* and *Bacteroides*, respectively (Supplementary Table S2). Furthermore, the genus *Prevotella* consisted mostly of *Prevotella copri*, whereas the genus *Bacteroides* contained several species (Supplementary Table S2).

The differences in family composition between the two cities were more pronounced between P-type and BB-type groups as shown in the right panel of **Figure 3A**. Genus and species compositions were also compared between the enterotype groups (Supplementary Table S2). The contrast between P- and BB-types was mostly similar as observed in the five countries in AMP Phase I study, while *Bacteroides* was more depleted in P-type samples from Leyte children. The trade-off between *Prevotella* and *Bacteroides* is common among the human gut microbiota variation, explaining two of the three enterotypes (Koren et al., 2013). Ruminococcaceae were slightly but significantly higher in Ormoc than in Baybay children. Bifidobacteriaceae and Lachnospiraceae did not show statistically significant differences both between cities and between enterotypes, although these two families were drivers for BB-type cluster in the AMP Phase-1 dataset together with Bacteroidaceae and Ruminococcaceae (Nakayama et al., 2015). Regarding subdominant group, *Succinivibrio* was markedly abundant and prevalent in the Leyte P-type samples (4.1% for abundance and 56% for prevalence) compared to Leyte BB-type (0.003% for abundance and 5.6% for prevalence) and P-type of other five countries (0.4% for abundance and 20% for prevalence).

Observed species richness represented by the number of detected OTUs per sample also differed statistically between the two enterotype groups, but not between the two cities (**Figures 3B,C**). Phylogenetic diversity represented by the index of PD_whole_tree showed the same trend as the observed species. Shannon–Wiener index did not show statistical difference both between cities and between enterotypes. The higher alpha-diversity in P type is previously reported in the AMP Phase-I study. Compared to other countries sampled in AMP Phase-I study, these Leyte children showed a middle level of alpha-diversities for those indices, around the same level as China and Thailand following Indonesian children (Supplementary Figure S4).

### Correlation between Dietary Nutrients and the Gut Bacterial Community in Leyte Children

**FIGURE 4:**
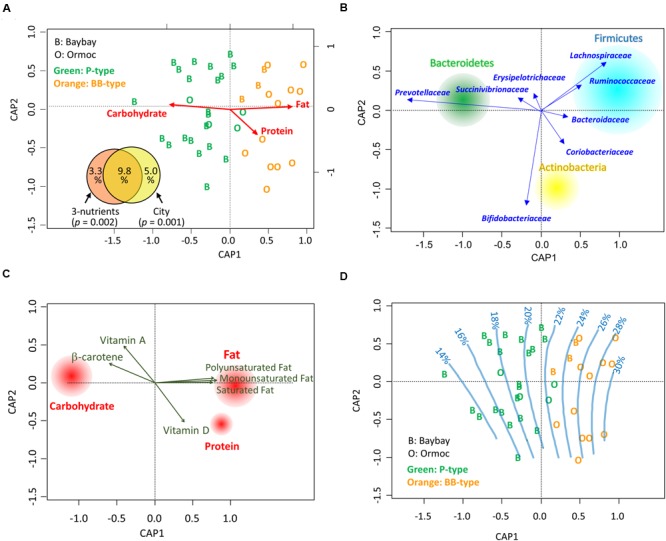
**Correlation between dietary nutrients and gut bacterial communities in Leyte children. (A)** Constrained analysis of principal coordinates (CAP) for the correlation between macronutrient intake and fecal bacterial composition. The individual datasets (*n* = 24 for Baybay, indicated by alphabet “B”; *n* = 16 for Ormoc, indicated by “O”) describing the energy ratios of macronutrient (carbohydrate, fat, and protein) intake were subjected to distance-based redundancy analysis (db-RDA) based on the Bray–Curtis distance between their family level bacterial communities. The alphabets representing the city of sample origin are colored according to the microbiota types classified in **Figure 2B**; “Green” and “Orange” letters indicate P-type and BB-type, respectively. The inset partition diagram explains bacterial community variance (adjusted *R*^2^) in terms of dietary macronutrients and/or city of residence, with *p*-values showing the significance of constraints calculated with 999 permutations. **(B)** Loading plot of bacterial phyla and families on the CAP ordination. The loadings of families were calculated by the db-RDA. The loadings of the three major phyla were calculated using envfit analysis to fit the phylum compositions of 40 samples to the CAP ordination. The size of each phylum circle represents total population size in the 40 Leyte samples. **(C)** Envfit plot of dietary nutrients on the CAP ordination. The daily intake level of each nutrient was correlated with sample variance in the CAP using envfit analysis; the nutrients showing significant correlation in 999 permutations (*p* < 0.05) were displayed by loading vectors. For macronutrients, their energy ratio data were subjected to envfit analysis and all three showed significance. The size of each macronutrient circle is proportional to *R*^2^ in the envfit analysis. **(D)** Gradient of dietary fat intake (energy %) on the CAP ordination. The fat energy intake ratio of the 40 Leyte children was fitted to the CAP coordinate by the ‘ordisurf’ function and regression splines are displayed.

To examine the correlation between macronutrient intake and the fecal bacterial community, we performed a constrained analysis of principal coordinates (CAP) using the db-RDA (Legendre and Anderson, 1999) on a dataset of 40 Leyte children. Variation in the fecal bacterial community correlated significantly with individual macronutrient intake variation (*p* = 0.002 in 999 permutation tests, **Figures 4A,B**). Even after constrained ordination, enterotype-like clusters were still apparent (green and orange letters in **Figures 4A,B**). Differences in macronutrient intake accounted for 13.1% of bacterial community variation, most (9.8%) of which was explained by the city of residence (Venn diagram in **Figure 4A**). This indicated that the discrepancy in gut microbiota could be ascribed to dietary differences associated with the city of residence.

Next, we performed an envfit analysis to examine the correlation between micro- and macronutrients and CAP-ordinated bacterial community variables (**Figure 4C**). The three macronutrients showed similar correlations as in the CAP (**Figure 4A**), with fat and carbohydrate being statistically significant (*p* < 0.001) and protein being marginally significant (*p* = 0.087). In addition, the three main constituents of fats and three vitamins also showed a significant correlation (*p* < 0.05). Fats favored BB-type microbiota, characterized by the high abundance of the phylum Firmicutes, and were oppose to P-type, characterized by the high abundance of the phylum Bacteroidetes (**Figure 4C**). On the other hand, β-carotene and vitamin A favored P-type bacteria (**Figure 4C**). The loadings of these two micronutrients mainly reflected a dietary habit of Baybay children who daily consume high amount of regional fruits, such as green mango and banana; Baybay children ate 109 g/d of green mango and 84 g/d of banana, whereas Ormoc children ate no green mango and 49 g/d of banana. Finally, we created an ordisurf plot to regress the fat intake level with the fecal bacterial community (**Figure 4D**). This shows the gradient in fat intake level from the cluster of the Baybay children harboring P-type microbiota to that of the Ormoc children harboring BB-type microbiota. Moreover, to examined the effect of two subjects administrated antibiotics on this constrained analysis, we performed same analysis by using the dataset excluding these two samples. As shown in Supplementary Figure S5, we obtained mostly same ordination. Therefore, we performed further analysis without removal of these two samples.

**FIGURE 5:**
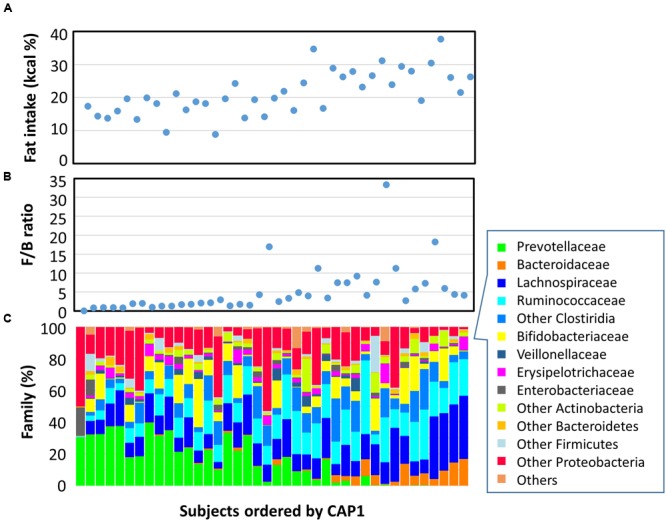
**Fecal bacterial composition and correlation with dietary fat intake.** Plots representing data from 40 Leyte children are ordered by CAP1 score on the *x*-axis, and by their fat energy intake ratio **(A)**, fecal Firmicutes-to-Bacteroidetes (F/B) ratio **(B)**, and fecal bacterial family composition **(C)** on the *y*-axis.

In **Figure 5**, Leyte children were sorted according to the score of the first component of the CAP coordinate (CAP1). We plotted their fat intake level (A), Firmicutes-to-Bacteroidetes (F/B) ratio (B), and family level bacterial composition (C). Graphs A and B indicated that fat intake and F/B ratio correlated with CAP1, suggesting a link between fat intake and F/B ratio in gut microbiota. Indeed, Spearman’s rank correlation analysis indicated a rho = 0.5824 (*p* = 0.0001) between F/B ratio and fat intake level in individuals. Graph C showed that Prevotellaceae’s abundance decreased with an increase in CAP1. Specifically, in the high CAP1 score range with high fat intake levels ranging from 20 to 40% of total energy intake indicated in graph C, Prevotellaceae were replaced by Bacteroidaceae. This suggested that a high-fat intake inhibited colonization and/or growth of Prevotellaceae and favored BB-type microbiota instead.

To examine the relationship between fat intake and gut commensals in more detail, we calculated Spearman’s correlation coefficient between fat energy intake rate and the relative abundance of each taxonomic group (**Table 2**). We confirmed that total fat intake correlated negatively with Bacteroidetes and positively with Firmicutes. Nevertheless, not all genera clustered equally. Thus, the genus *Prevotella* correlated negatively with fat intake (rho = -0.6315; CI95%: -0.79 to -0.40; *p* < 0.0001), whereas the genus *Bacteroides* correlated positively with fat intake (rho = 0.5298; CI95%: 0.26 to 0.72; *p* = 0.0004). In Firmicutes, the order Clostridiales showed significant positive correlation with total fat intake (rho = 0.5443; CI95%: 0.28 to 0.73; *p* = 0.0003), but the correlation became less clear for lower taxonomic ranks, suggesting that not a specific but a broad range of Clostridiales favored high fat. Genus *Succinivibrio*, from the phylum Proteobacteria, showed a negative correlation with total fat intake (rho = -0.350; CI95%: -0.60 to -0.04; *p* = 0.0267). As well as *Prevotella, Succinivibrio* is known as a plant polysaccharide-fermenting bacterium overrepresented in the *Prevotella*-type gut microbial community of native Africans (Ou et al., 2013).

**Table 2:** Spearman’s rank correlation coefficient between fat intake ratio and relative abundance of each taxonomic group.

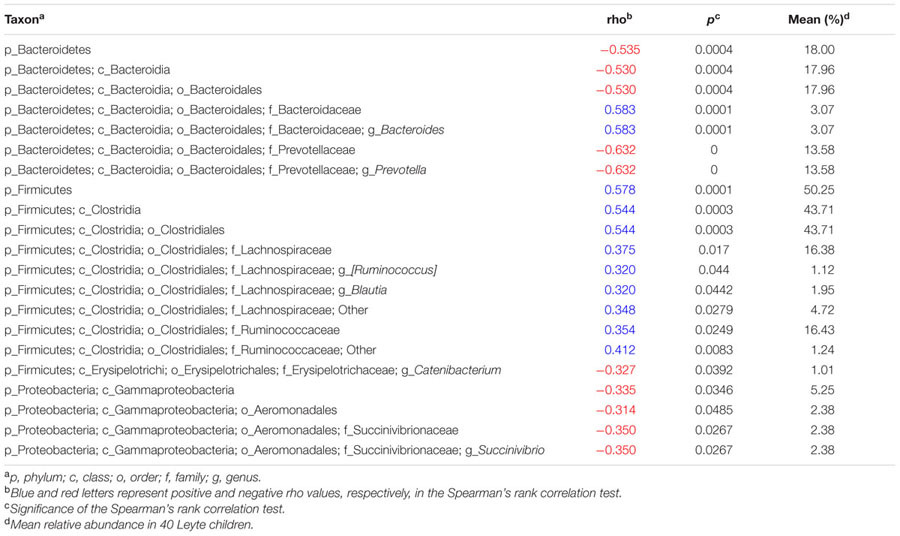

**FIGURE 6:**
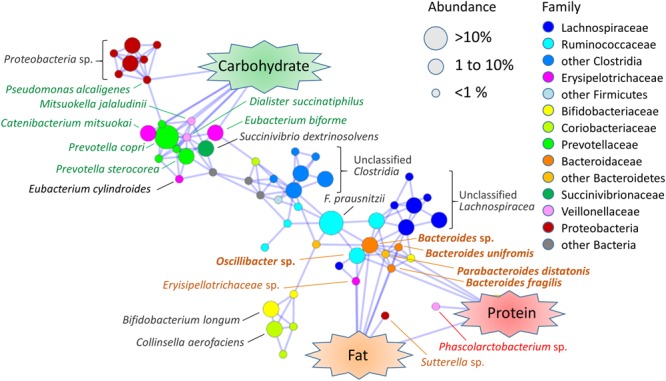
**Association map among fecal bacterial species and dietary macronutrients in Leyte children.** Pairwise Spearman’s correlation analysis was performed using the 40 Leyte children dataset of the relative abundances of 80 species (mean % > 0.1% for 40 children samples) and the energy ratio of the daily consumed three macronutrients. Correlation coefficients higher than 0.4 or lower than -0.4 with *p* < 0.05 were extracted and are visualized in association map using the Prefuse Force Directed Layout in Cytoscape 3.3.0. Only positive correlations are represented as blue-line edges. The colors of the species nodes represent taxonomy at family or higher level. The sizes of nodes represent mean population among the Leyte children. Species name associated with nodes indicates a closest species identified by SeqmatchQ400 analysis. Species names colored green, orange, or red represent species showing a significant association with the intake ratio of carbohydrate, fat, and protein, respectively. Species names colored orange and bolded represent species showing a significant association with intake ratio of both fat and protein.

To deepened our insight about the association of gut microbes with dietary nutrients, we analyzed total correlation among the abundance of fecal bacterial species and macronutrients consumption and created its association map (**Figure 6**). As observed in the phylum-to-genus level analysis (**Table 2**), *Bacteroides* species associated with fat/protein and *Prevotella* species associated with carbohydrate. Furthermore, two *Erysipelotrichaceae* species, related to *Eubacterium biforme* and *Catenibacterium mitsuokai*, and two *Veillonellaceae* species, related to *Mitsuokella jalaludinii* and *Dialister succinatiphilus*, showed positive correlation to dietary carbohydrate level. This finding coincides with previous reports showing that *M. jalaludinii* was decreased by high-fat sucrose diet (Etxeberria et al., 2015) and that *C. mitsuokai* was induced in pig cecum by a dietary fiber (Yan et al., 2013). *Succinivibrio dextrinosolvens* was associated with these carbohydrate-associated bacteria groups, although it was not directly correlated with dietary carbohydrate. *S. dextrinosolvens* is known to fermentatively produce succinate as an endproduct (O’Herrin and Kenealy, 1993), as well as *Prevotella* species. *D. succinatiphilus*, which is known as a succinate-loving species whose growth is enhanced by the addition of succinate (Morotomi et al., 2008), is located at the center of these succinate-producing bacteria community. This suggests that there is a syntrophy via succinate within this carbohydrate-associated community, although further studies on molecular level are warranted.

Dietary fat and protein commonly associated with a number of species belonging to genera *Bacteroides, Parabacteroides* and *Oscillibacter.* An identified *Phascolarctobacterium* species was associated only with protein. Unidentified *Sutterella* species and *Erysipelotrichaceae* species were associated only with fat. Genera *Oscillibacter* and *Phascolarctobacterium* and family *Erysipelotrichaceae* were reported to be increased by high fat diet (Lam et al., 2012; Etxeberria et al., 2015; Lecomte et al., 2015) It is noted that, except for *Oscillibacter*, no *Clostridiales* species directly correlated with dietary fat, although order *Clostridiales* show strong positive correlation with fat consumption as shown in **Table 2**. However, many *Lachnospiraceae* species (blue nodes in **Figure 6**) and *Ruminococcaceae* species (light blue nodes in **Figure 6**) were closely associated with the fat-associated *Bacteroidaceae* community. This indirect association of large number of *Clostridiales* species may explain strong positive correlation of *Firmicutes*-*Clostridiales* with fat consumption.

**FIGURE 7:**
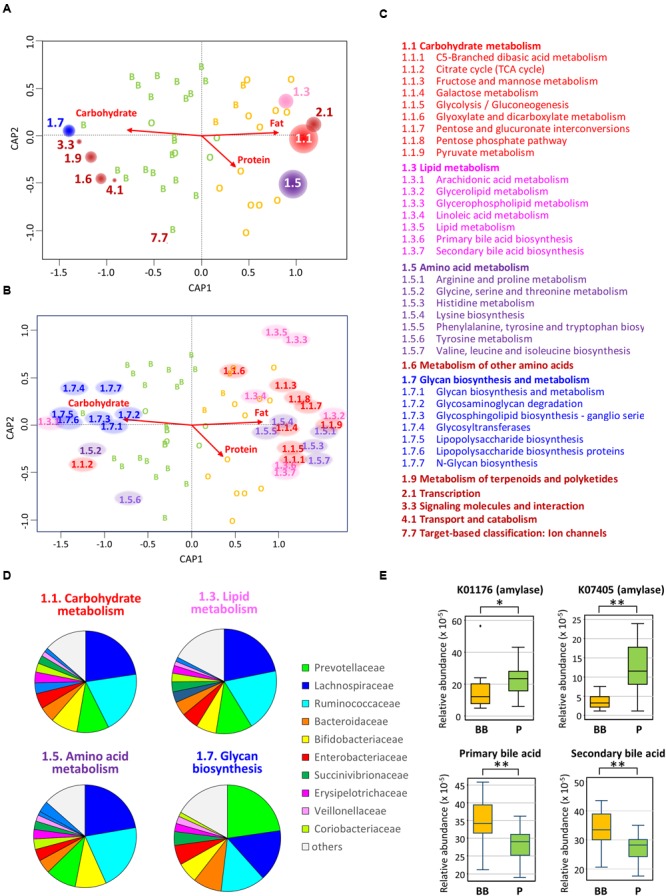
**Correlation between gut bacterial community and genome-encoded functions. (A)** Envfit plot of KEGG pathways at hierarchy level 2 on the CAP ordination computed in **Figure 4**. The abundances of KEGG pathways at hierarchy level 2 was estimated in each subject by the PICRUSt analysis based on the 16S rRNA composition data. Then, they were correlated with sample variance in the CAP by using envfit program; the KEGG pathways showing significant correlation in 999 permutations (*p* < 0.05) were displayed by red circles placed at the tips of undrawn vectors. The size of circle for each KEGG pathway is proportional to *R*^2^ in the envfit analysis. The code number of KEGG pathway is written beside the circle and is listed with the functional annotation in **(C)**. **(B)** Envfit plot of KEGG pathway at hierarchy level 3 on the CAP ordination. The plot was simulated by the same methods used for level 2 in **(A)**. The KEGG pathways showing significant correlation in 999 permutations (*p* < 0.05) were displayed by the pathway code no. placed at the tips of undrawn vectors. The code numbers are colored by the KEGG pathway hierarchy level 2 and are listed with the functional annotation in **(C)**. **(C)** The code numbers and their functional annotations of KEGG pathways figured in **(A)** for hierarchy level 2 and **(B)** for hierarchy level 3. The letters are colored the same as for the codes in the other panels. **(D)** Contribution of each bacterial family to the KEGG pathways in the bacterial community of Leyte children. For each KEGG pathways, gene counts were estimated in each bacterial family based on the KEGG gene content table (ko_13_5). Then, counts from all families were summed and graphed as pie chart. **(E)** Abundances of two KEGG genes annotated as amylases (upper two graphs) and KEGG pathways, respectively, annotated as primary and secondary bile acids (lower two graphs). Relative abundances of these KEGG genes and KEGG pathways were calculated by the PICRUSt and were displayed as box plots showing the smallest and largest values, 25 and 75% quartiles, the median, and outliers. Statistical difference in the abundance was examined between BB-type and P-type groups by Wilcoxon rank-sum test for the two amylase genes and Student’s *t*-test for the pathways of primary and secondary bile acid biosynthesis. Single and double asterisks represent *p* < 0.05 and *p* < 0.001, respectively.

To understand the functional link between the dietary change and the bacterial community shift in Leyte children, we employed PICRUSt to predict abundance of functional genes in each subject. **Figures 7A–C** shows that genes involved in metabolism of sugar, lipid, and amino acids are enriched in BB-type microbiota, while genes involved in glycan biosynthesis and metabolism are overrepresented in P-type microbiota. It was indicated that this contrast is largely driven by Prevotellaceae that is the major driver of P-type (**Figure 7E**). Two KEGG genes annotated as amylase were predicted to be more abundant in P-type samples than BB-type samples (upper two graphs in **Figure 7E**). Furthermore, two KEGG pathways, respectively, annotated as primary and secondary bile acid biosynthesis were predicted to be more abundant in BB-type samples than P-type samples (lower two graphs in **Figure 7E**).

### Link between High-Fat Diet-Altered Gut Microbiota and Obesity in Leyte Children

**FIGURE 8:**
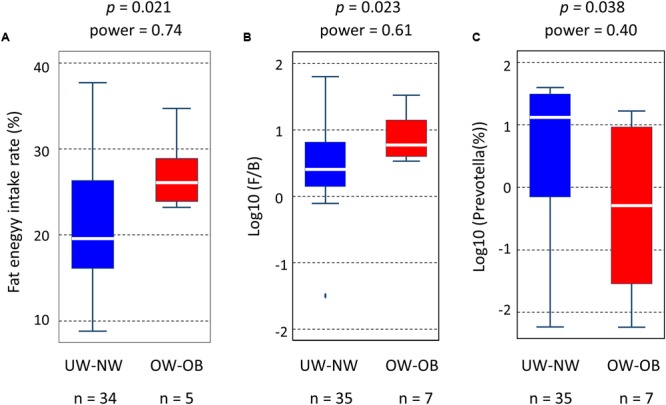
**Correlation between high-fat diet-altered gut microbiota and obesity in Leyte children. (A)** Distribution of fat energy intake (%), **(B)** Firmicutes-to-Bacteroidetes (F/B) ratio, and **(C)** relative abundance of *Prevotella* in underweight (UW) – normal weight (NW) group and overweight (OW) – obese (OB) group. Statistical differences between the two groups were examined by the Student’*t*-test in **(A)** and the Wilcoxon rank-sum test in **(B)** and **(C)**. The *post hoc* power analysis was perform to retrospectively examine the observed power in these tests.

Body mass index (BMI) values among children of Leyte island (16.5 ± 3.8 for boys; 16.5 ± 3.6 for girls) were comparable to those of age-matched individuals in the United States (Body Mass Index: BMI for Children and Teens. Centers for Disease Control and Prevention. Retrieved 2013-12-16^11^). Four children were categorized as overweight [BMI from 19.2 kg/m^2^ (85th percentile) to 25.6 kg/m^2^ (95th percentile)] and three as obese [BMI greater than 25.6 kg/m^2^ (95th percentile)] (Wang and Lim, 2012). Interestingly, all overweight and obese children were living in Ormoc, suggesting a link between this condition and modern high-fat dietary habits. On the other hand, four children from Baybay and one from Ormoc were categorized as underweight [BMI lower than 13.75 kg/m^2^ (15th percentile)]. Daily fat intake was significantly higher in the overweight-obese group than in the normal-underweight group (**Figure 8A**), whereas total energy intake did not statistically differ between the two groups (Supplementary Table S1). Moreover, as shown in **Figures 8B,C**, F/B ratio and relative abundance of *Prevotella* were significantly higher and lower, respectively, in the overweight-obese group than in the normal-underweight group, although the observed power determined retrospectively was not statistically high enough to warrant significance. On the other hand, there was no significant difference in daily fat intake, F/B ratio, or *Prevotella* population between normal-weight and underweight groups (data not shown). The correlation between altered gut microbiota and high BMI suggests that high-fat diet-associated obesity is present among Filipino children on Leyte island.

## Discussion

Variations in the gut bacterial community of Leyte children co-clustered with those of other Asian children. The commonality and robustness of microbiota typing, which was largely defined by the presence or absence of Prevotellaceae, suggest the existence of common factor(s) driving either microbial community. It is also noted that some other species associate with the core genera of each type, namely *Prevotella* and *Bacteroides*, and these species-to-species and species-to-diet fine networks may stabilize the each enterotype-like community. Among Leyte children, the occurrence of these two types of microbial communities was linked to a diet-dependent nutrient bias, in which the high-fat diet of Ormoc children strongly limited P-type microbiota. It is known that a high-fat intake increases the intestinal level of bile acids, including highly toxic secondary bile acids, resulting in fewer bile-sensitive bacteria and more bile-tolerant bacteria (Rafter et al., 1987; Islam et al., 2011; David et al., 2013). This may explain the fact that *Prevotella*, known for being sensitive to bile acid, was less prevalent in BB-type communities. The present predictive metagenomics using PICRUSt indeed indicated the overrepresentation of both primary and secondary bile acid biosyntheses in P-type samples (**Figure 7E**).

In contrast to fat, plant carbohydrates such as dietary fibers have been recognized as a major driver of *Prevotella*-type microbiota (De Filippo et al., 2010; Wu et al., 2011; David et al., 2013; Durbán et al., 2013; Glick-Bauer and Yeh, 2014; Matijašić et al., 2014; Holscher et al., 2015; Kovatcheva-Datchary et al., 2015; Martínez et al., 2015). Further, there is a suggestion that non-digestible starch could be a determining factor for P-type (Nakayama et al., 2015). In this study, the PICRUSt predicted the enrichment of amylase genes in P-type samples, suggesting overrepresentation of non-digestible starch in the intestine of P-type subjects. However, we could not find statistical difference or correlation with the estimated intake of dietary fibers. Instead, we found the positive correlation between β-carotene/vitamin A and P-type microbiota. Leyte children ingested these vitamins mainly from regional fruits, such as green mango and banana, which are also known to contain high amount of dietary fibers. Detailed dietary investigations may address the questions regarding plant dietary factors that promote P-type microbiota in children not only on Leyte island but also over the Southeast Asia.

The PICRUSt analysis has further suggested the difference in the metabolic activity between the two types of microbiota. It appears that BB-type microbiota is well nourished and metabolically more active with simple sugars, amino acids, and lipids, while P-type is more involved in digestion of complex carbohydrate. This functional contrast reflects well the dietary record investigated in this study. The enrichment of KEGG pathways involved in simple sugar metabolism was also previously reported in Western diet-associated microbiome of the obesity-induced mice (Turnbaugh et al., 2008). Further, the same group reported the enrichment of glycan metabolism pathway in the microbiome of humanized mice fed a low-fat and plant polysaccharide-rich diet (Turnbaugh et al., 2009).

A number of studies have described the link between gut microbiota and obesity; some have demonstrated a role for certain commensals in the harvest and storage of the energy derived from ingested nutrients (Bäckhed et al., 2004; Turnbaugh et al., 2006; Bäckhed et al., 2007). In particular, the higher F/B ratio associated with obesity has been highlighted (Ley et al., 2006; Turnbaugh et al., 2008; Turnbaugh et al., 2009; Kasai et al., 2015), although the results were not always consistent among studies and there is controversy about the causality among diet, gut microbiota, and obesity (Duncan et al., 2008; Harley and Karp, 2012). To this end, recent studies has demonstrated that altered gut microbiota with an increased F/B ratio promotes diet-induced obesity by affecting the bile acid profile that modulates host metabolism via the intestinal farnesoid X receptor (Li et al., 2013; Parséus et al., 2016). In the present study, regarding *Firmicutes*, we found two directly fat-associated species, namely *Oscillibacter* sp and *Erysipelotrichaceae* sp. and a number of indirectly fat-associated *Clostridiales* species. The high F/B gut microbial community consisting of these species might accelerate a modern-diet associated obesity. For instance, *Oscillibacter*-like bacteria are known as a potentially important gut microbe that mediates high-fat diet-induced gut dysfunction (Lam et al., 2012).

Bacteroidetes inhabiting P-type children on Leyte island belonged mostly to the genus *Prevotella.* The relative abundance of *Prevotella* inversely correlated with fat intake and BMI among Leyte children. This agrees with a previous paper by Furet et al. (2010), whereas some papers have oppositely reported higher level of *Prevotella* in obese subjects (Zhang et al., 2009; Durbán et al., 2012; Hu et al., 2015). It is known that H_2_-producing *Prevotella* coexists with relatively high numbers of H_2_-utilizing methanogenic Archaea in the gastrointestinal tract of obese persons (Samuel and Gordon, 2006; Zhang et al., 2009; DiBaise et al., 2012). This has suggested that hydrogen transfer between bacterial and archaeal species accelerates short chain fatty acids fermentation by *Prevotella*, resulting in enhancement of energy production in intestine. Although this syntrophism is not beneficial for the obese persons with sufficient body energy, it may play a crucial role for energy harvest in individuals with less nutrient supply. In fact, previous studies have observed that proportion of *Bacteroides*-*Prevotella* group increased under a starvation-like condition after gastric bypass surgery (Nadal et al., 2009; Furet et al., 2010).

In the present study, calorie intake level of children in Baybay was less than those in Ormoc although it was not statistically significant (Supplementary Table S1; Supplementary Figure S3). Further, weight and height of Baybay children were significantly lower, compared to Ormoc children (**Table 1**) and average among developed countries, although the levels ranged within normal limits indicated by the World Health Organization. These data suggest a marginal energy restriction in Baybay children. The previous paper described that *Prevotella*-rich gut microbiota of Burkina Faso children produced a higher level of short chain fatty acids than Firmicutes-rich microbiota of Italian children, suggesting efficient energy harvest from calorie-less indigestible dietary fibers (De Filippo et al., 2010). It has been also found that native African had higher concentrations of stool short chain fatty acids than African American (Ou et al., 2013). The study reported that *Succinivibrio* which utilizes H_2_ to reduce sulfate to hydrogen sulfide was overrepresented together with H_2_-utilizing methanogen in the native African and the higher rate of short chain fatty acid fermentation may be due to the syntrophism between *Prevotella* and those H_2_-utilizing micro-organisms. The present study also found that *Succinivibrio* was overrepresented in association with in the P-type microbiota of Leyte children, although methanogens were not detected here due to the used primers targeting eubacteria. It is further interesting to note the possibility of a succinate-mediated syntrophy in the P-type community including *Prevotella, Succinivibrio*, and some other subdominant species such as *D. succinatiphilus* known as succinate-utilizing bacterium.

Recently, a translational study has demonstrated that barley kernels promote glycogen storage and improve glucose metabolism by increasing *Prevotella* (Kovatcheva-Datchary et al., 2015). In the animal experiment, glucose tolerance was impaired in mice colonized with *Bacteroides thetaiotaomicron* and was rescued by co-colonization with *P. copri.* Taken together, *Prevotella* appears to play a pivotal role in the beneficial response to less calorie diets. We can see, then, that the *Prevotella*-depleted high F/B gut microbiota, corresponding to P-enterotype, is the result of evolutionary adaptation of gut microbial community to high-fat and high-calorie modern diets.

Although we found the correlation between obesity and enterotype in this study, it is yet to be answered whether the altered gut microbiota is a cause of obesity or just a consequence of altered dietary habit. It is also noted that this study has limitations due to relatively small sample size and lack of in-depth biological and biochemical information, not allowing to address the complex physiological link among diets, microbes, host, and other underlying factors. However, this study suggests that the altered gut microbiota would be a sign of modern-diet associated obesity among children in developing areas. Since childhood obesity can lead to a variety of adverse health outcomes and associate with life style-related diseases in adulthood, AMP will keep an eye on gut microbiota of Asian children, especially in urbanizing regions.

## Ethics Statement

The ethics committee of the Faculty of Agriculture of Kyushu University The NUS Institutional Review Board Written informed consent was obtained from the parents/guardians of all participants.

## Author Contributions

Conceived and designed the experiments: JN, KS, JT, and Y-KL. Performed the experiments: JN, AY, and KH. Sampling: LP-C and JT. Analyzed the data: JN, LP-C, AY, and KH. Wrote the paper: JN, JT, and Y-KL.

## Conflict of Interest Statement

The authors declare that the research was conducted in the absence of any commercial or financial relationships that could be construed as a potential conflict of interest.

## Footnotes

1 http://qiime.org/

2 http://rdp.cme.msu.edu/seqmatch/seqmatch_intro.jsp

3 http://enterotype.embl.de/enterotypes.html

4 http://cran.r-project.org/web/packages/clusterSim/index.html

5 http://cran.r-project.org/web/packages/ade4/index.html

6 https://cran.r-project.org/web/packages/vegan/

7 http://www.mothur.org/

8 http://www.cytoscape.org/

9 http://huttenhower.sph.harvard.edu/galaxy/root, version 1.0.0

10 http://www.genome.jp/kegg/pathway.html

11 http://www.cdc.gov/healthyweight/assessing/bmi/childrens_bmi/about_childrens_bmi.html
