# Supplementary material for: Impact of Westernized Diet on Gut Microbiota in Children on Leyte Island
Source: Front Microbiol. 2017 Feb 14;8:197. doi: 10.3389/fmicb.2017.00197 (PMC5306386; doi:10.3389/fmicb.2017.00197)
Supplement: Supplementary file 1 [file Data_Sheet_1.pdf]

Supplementary Figures and Tables

Impact of High-Fat Modern Diet on Gut Microbiota in Children on Leyte Island

Jiro Nakayama[1], Azusa Yamamoto[1], Ladie Anne Palermo-Conde[2], Kanako Higashi1, Kenji Sonomoto1, Julie Tan[2], Yuan-Kun Lee[3]*

[1]Laboratory of Microbial Technology, Department of Bioscience and Biotechnology, Faculty of Agriculture, Kyushu University, Fukuoka, Japan
[2]PhilRootcrops, Visayas State University, Baybay City, Philippines
[3]Department of Microbiology, National University of Singapore, Singapore, Singapore

*Correspondence:
Yuan-Kun Lee
yuan_kun_lee@nuhs.edu.sg

P2: Supplementary Figure S1. Distribution of energy and nutrient intake level among the 43 Leyte children.
P3: Supplementary Figure S2. Variation of consumed foods and their energy among Leyte children (Baybay n=24, Ormoc n=16).
P4: Supplementary Figure S3. Levels of consumed amount and energy ratio of three macronutrients in Baybay (n=24) and Ormoc (n=16) children.
P5: Supplementary Figure S4. Alpha-diversities of fecal bacterial community in individual samples from school-age children from six countries in Asia.
P6: Supplementary Figure S5. The constrained analysis of principal coordinates (CAP) to correlate macronutrient profile with gut bacterial community variation using the Leyte children dataset with and without subjects administrated antibiotics
P7: Supplementary Table S1. Daily intake of energy and nutrients in children in Baybay, Ormoc and Japan.
P8: Supplementary Table S2. Genera and species showing significant difference in abundance between P- and BB-type samples

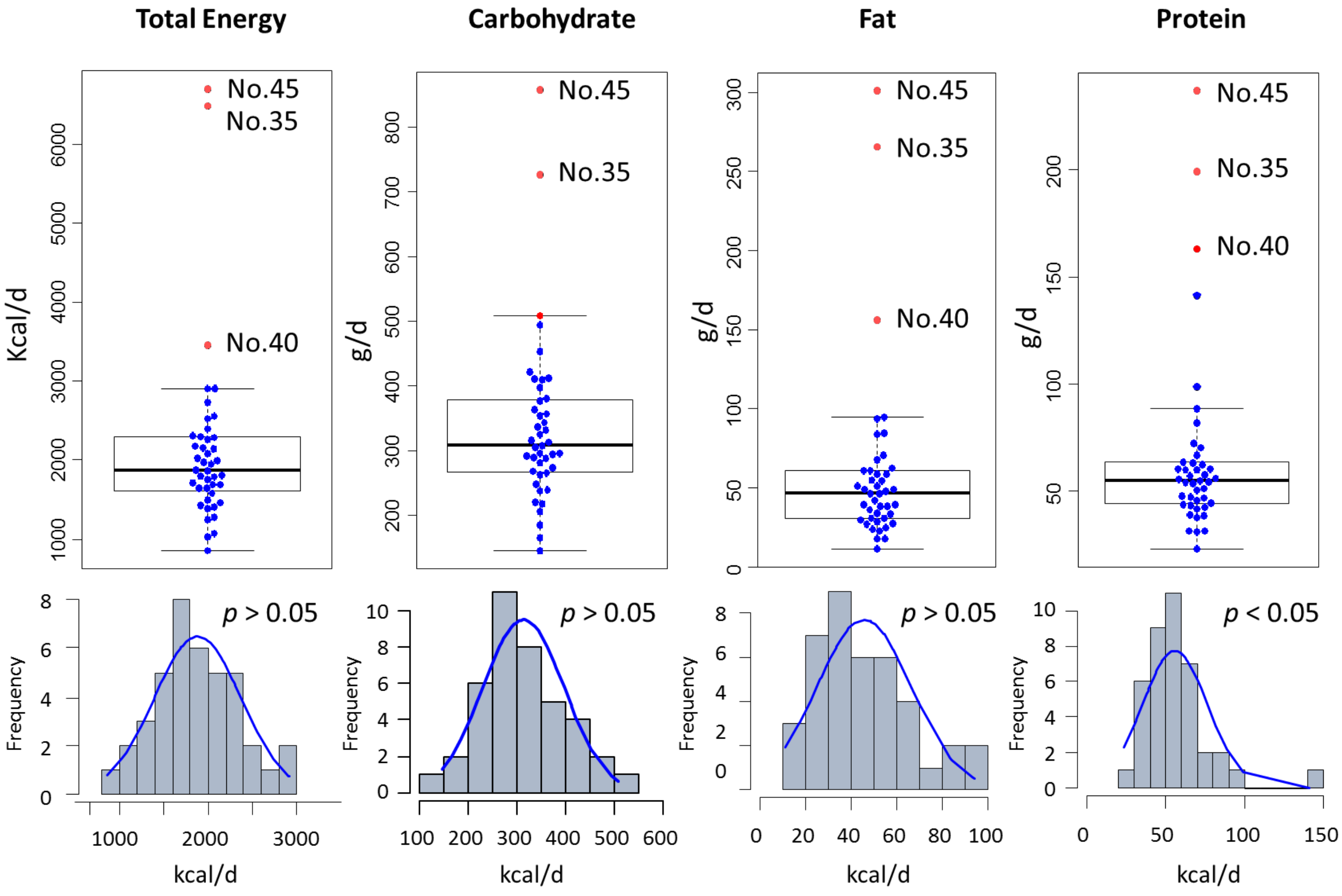

Supplementary Figure S1. Distribution of energy and nutrient intake level among the 43 Leyte children. Upper panels represent scatter graph overlaid on box plots showing the smallest and largest values, 25% and 75% quartiles, the median, and outliers. Red dots represent the samples recognized as outliers according to the maximum normed residual test (grubb's test) using the data of total energy intake of the 43 subjects. Lower panels represent a histogram and normal distribution curve calculated from the mean and variance of the 40 subjects data excluding the three outliers. We performed Shapiro-wilk test to calculated *p* value for normality.

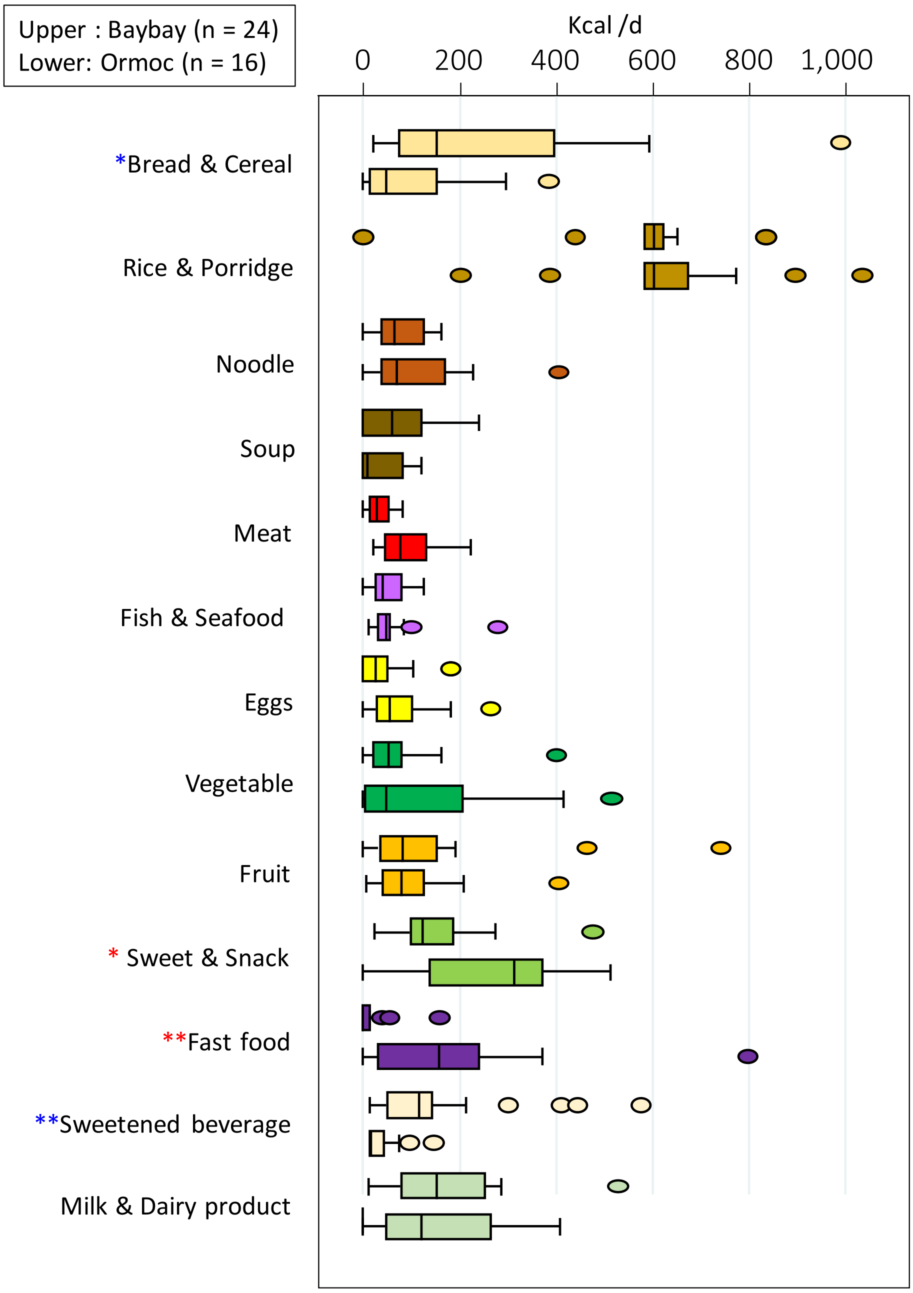

Supplementary Figure S2. Variation of consumed foods and their energy among Leyte children (Baybay n=24, Ormoc n=16). Daily consumption of each food was surveyed by the food frequency questionnaire (FFQ) to parent and was converted to energy units (kcal) according to the databases of energy and nutrition composition of food prepared by the Ministry of Health of Singapore and the Food and Nutrition Research Institute of the Philippines. In each food, upper and lower bars indicate Baybay and Ormoc children respectively. Box plot showing the smallest and largest values, 25% and 75% quartiles, the median, and outliers. Statistical difference was examined by Wilcoxon rank-sum test between Baybay and Ormoc groups and statistical significant was indicated by asterisk before food name. Red and blue asterisks indicate significantly higher in Baybay and Ormoc children, respectively, with $p < 0.05$ (single asterisk) or $p < 0.001$ (double asterisks).

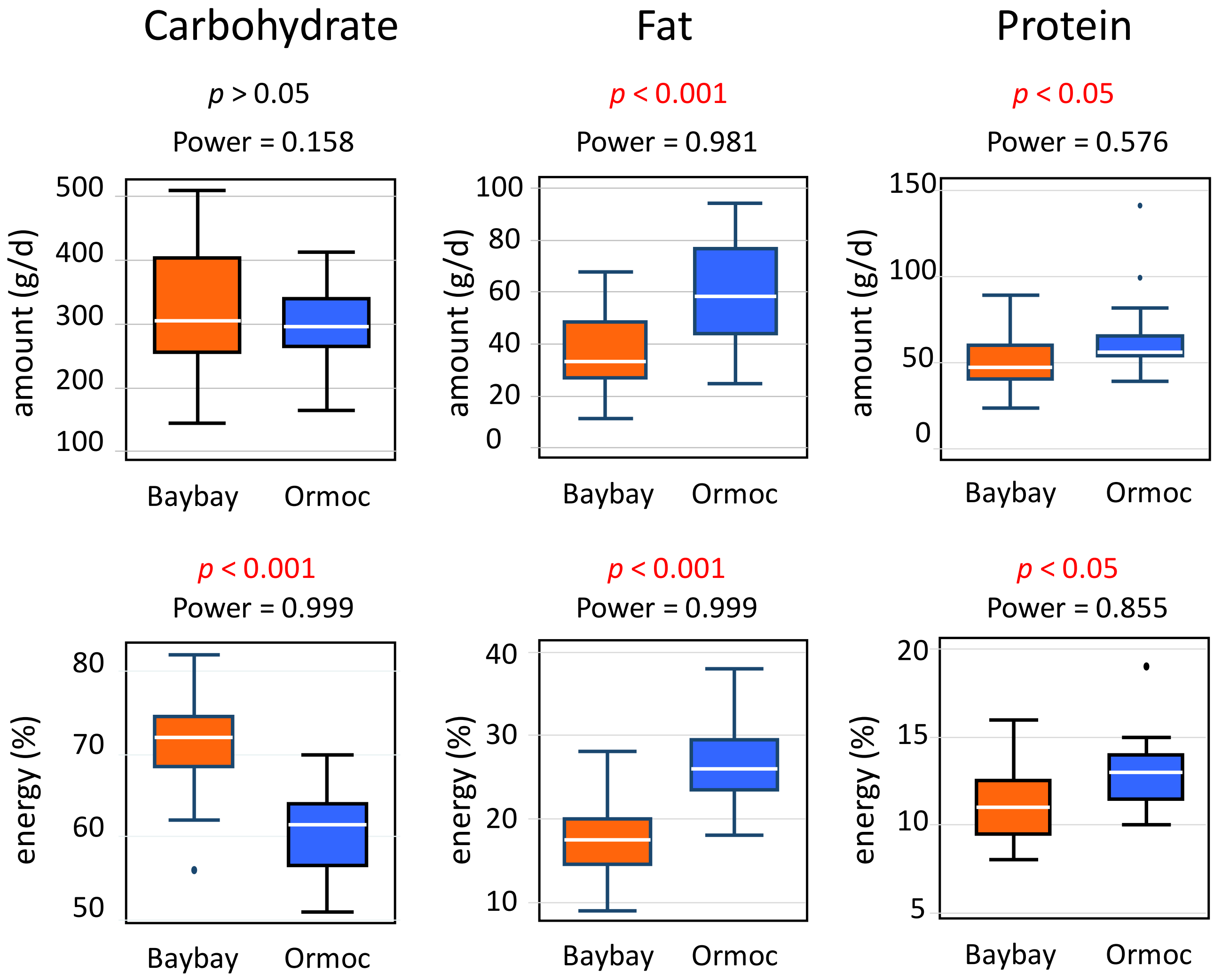

Supplementary Figure S3. Levels of consumed amount and energy ratio of three macronutrients in Baybay (n=24) and Ormoc (n=16) children. The consumed amount (g) of three macronutrients were estimated according to the food frequency questionnaire (FFQ) to the parents of participating children [Baybay (n =24) and Ormoc (n = 16)] and was converted to energy units (kcal) according to the database of energy and nutrition composition. The data of 40 children were represented by the box plots showing the smallest and largest values, 25% and 75% quartiles, the median, and outliers. Since data were normally distributed except for the amount of protein, all data were statistically compared by Student's *t*-test. Subsequently, a post-hoc power analysis was performed to retrospectively examine the observed power in these tests.

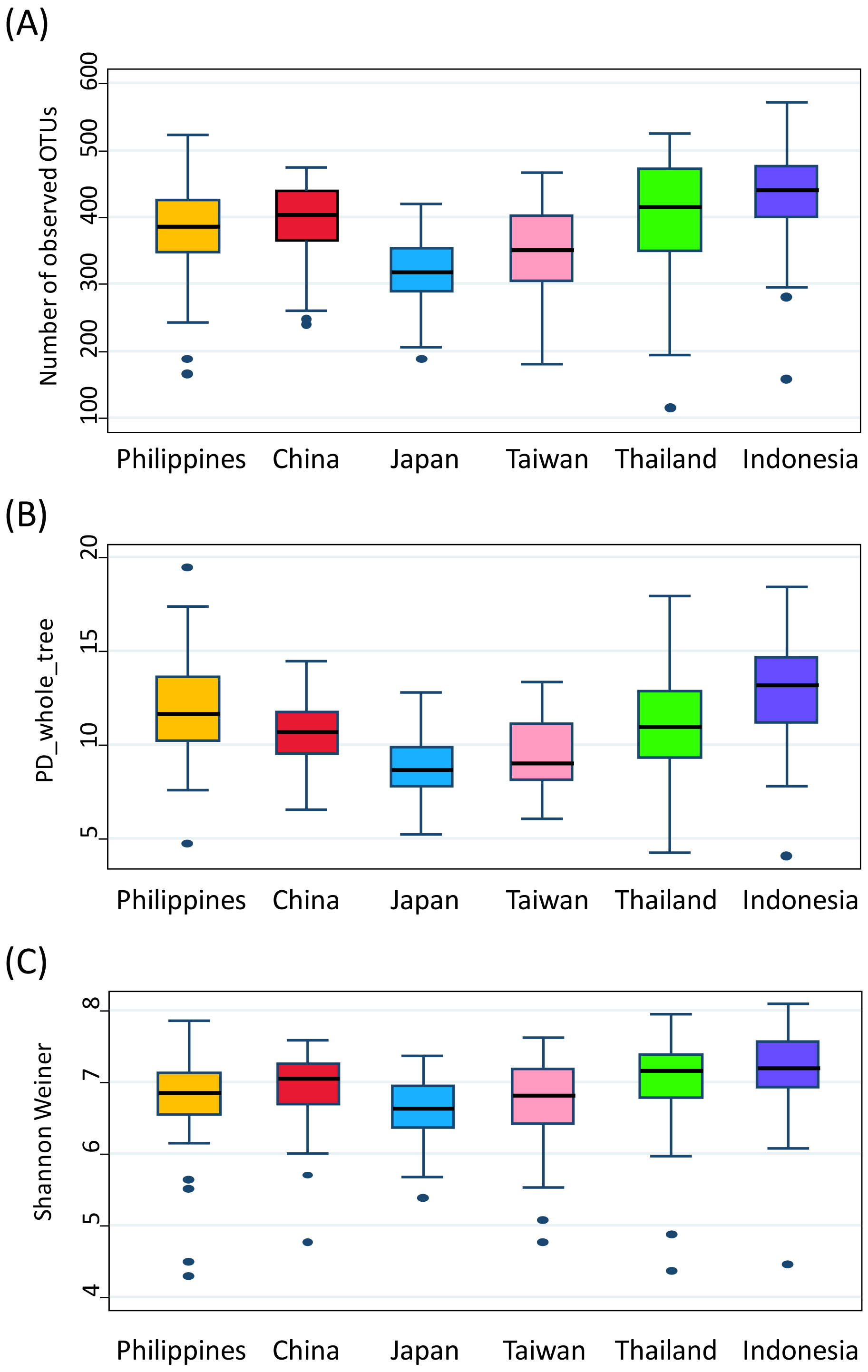

Supplementary Figure S4. Alpha-diversities of fecal bacterial community in individual samples from school-age children from six countries in Asia. Individual OTU-composition data (OTU table) were rarified using 2,000 reads per participant in ten iterations. The number of observed OTUs, PD_whole_tree, and Shannon Wiener index were calculated for each rarified OTU composition and averaged within the ten iterations. The covariance of these calculated indices was computed for each country and is graphed as a box plot showing the smallest and largest values, 25% and 75% quartiles, the median, and outliers.

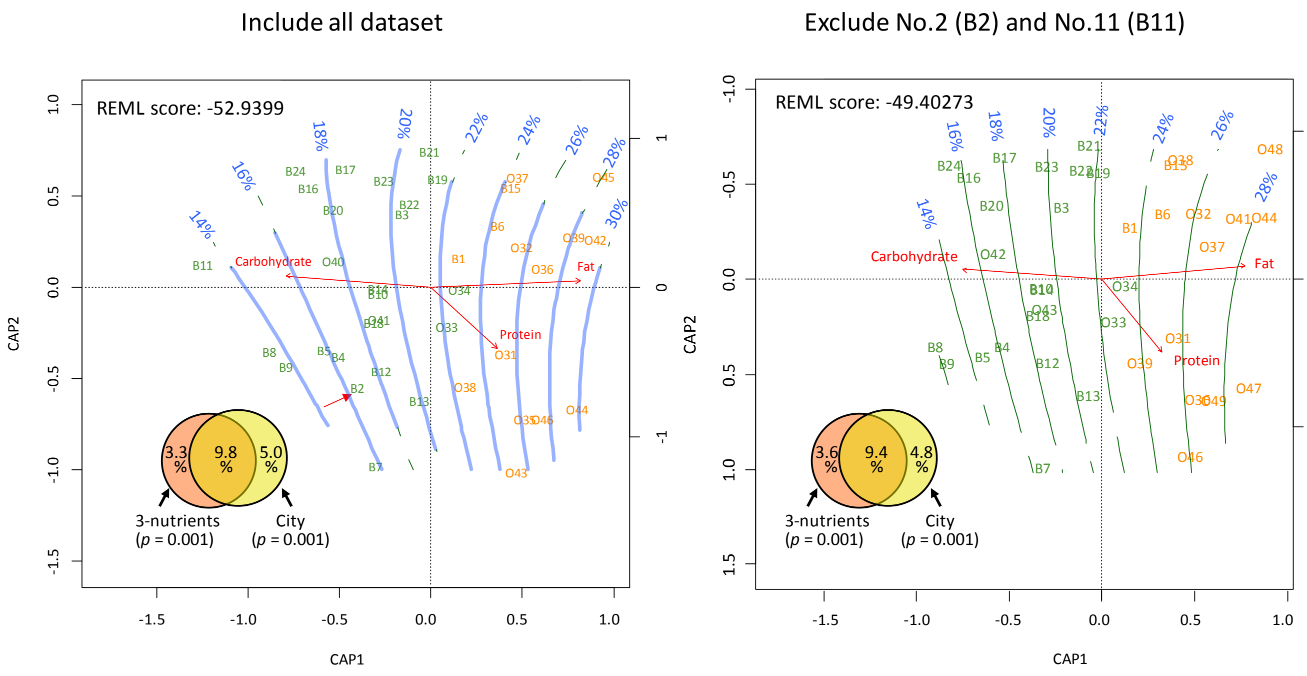

Supplementary Figure S5. The constrained analysis of principal coordinates (CAP) to correlate macronutrient profile with gut bacterial community variation using the Leyte children dataset with and without subjects administrated antibiotics. In the left CAP, the analysis was performed according to the same methods and dataset used in the analysis of Figure 4. In the right CAP, two samples from No. 2 (B2) and No. 11 (B11), who administration antibiotics within two weeks prior to sampling, were excluded and subjected to the same analysis.

Table 1. Daily intake of energy and nutrients in children in Baybay, Ormoc and Japan

	Baybay (n=24)		Ormoc (n = 16)				Japan[a]
	mean	SD	mean	SD	p [b]		mean
Energy (kcal)	1806.4	497.1	1987.9	482.4	0.2245		1963.9
Carbohydrate (kcal%)	71.6	6.0	60.4	6.0	<0.0001	***	56.5
Total (g)	323.3	93.6	297.6	67.2	0.5254		269.2
Starch (g)	199.9	54.3	199.8	46.7	0.5254		nd
Sugar (g)	83.1	60.5	54.0	16.5	0.0922		nd
Dietary Fiber (g)	17.9	9.5	15.5	3.7	0.9121		13.0
Fat (kcal %)	17.9	4.7	26.8	5.2	<0.0001	***	29.0
Total (g)	36.3	14.3	60.1	20.8	0.0001	**	63.8
Saturated (g)	15.9	5.9	29.2	10.7	0.0002	**	19.72
Monounsaturated (g)	13.5	5.7	22.0	8.1	0.0010	**	21.35
Polyunsaturated (g)	4.69	1.63	8.94	4.24	0.0008	**	nd
Cholesterol (g)	0.18	0.14	0.26	0.15	0.0140	*	0.32
Linoleic acid (g)	1.13	0.87	1.13	0.57	0.5254		nd
Alpha linolenic acid (mg)	35.9	70.9	70.5	62.2	0.0063	*	nd
Trans fatty acid (mg)	4.72	18.84	0.34	0.22	0.0272	*	nd
EPA.DHA	0.04	0.03	0.09	0.13	0.1176		nd
Protein (kcal)	11.2	2.2	12.9	2.3	0.0258	*	14.4
Total (g)	49.9	15.2	64.6	24.8	0.0214	*	70.6
Vitamin							
Vitamin A (mg)	1.40	0.88	1.04	0.84	0.1944		0.52
Retinol (mg)	0.80	0.56	0.75	0.40	0.8684		nd
b-Carotene (mg)	4.36	3.25	2.56	4.01	0.0219	*	nd
Thiamin (mg)	1.28	0.55	1.22	0.47	0.7614		0.90
Riboflavin (mg)	1.26	0.56	1.81	1.32	0.1156		5.06
Vitamin C (mg)	28.1	30.27	57.36	39.33	0.0058	*	68.7
VitaminD (μg)	0.37	0.34	0.79	0.56	0.0130	*	5.5
Mineral							
Sodium(g)	3.18	0.76	3.84	1.08	0.0684		3.48
Calcium (g)	0.58	0.24	0.73	0.25	0.0500		0.62
Potassium (g)	1.84	0.96	1.76	0.53	0.8468		2.13
Phosphorus (g)	0.74	0.24	0.94	0.27	0.0087	*	1.05
Iron (mg)	15.09	5.08	15.52	5.59	1.0000		6.57
Zinc (mg)	5.95	1.76	8.24	2.64	0.0024	*	8.67
Niacin (mg)	8.78	3.16	7.97	3.65	0.1761		12.0
Selenium (mg)	0.11	0.05	0.36	0.51	0.0163	*	nd

[a]Data from National Health and Nutrition Survey, Japan in 2014 (n=620) (http://www.mhlw.go.jp/seisakunitsuite/bunya/kenkou_iryou/kenkou/kenkounippon21/en/eiyouchousa/kekka_eiyou_chousa_nendo.html). "nd" means "no data".

[b]Difference between Baybay and Ormoc is calculated by Wilcoxon rank-sum test except for macronutrients, which Student t test was applied. p < 0.05, p < 0.001, p < 0.0001 are marked by "*", "**", and "***", respectively.

Supplementary Table S2. Genera and species showing significant difference in abundance between P- and BB-type samples.

Taxonomy[a]		Rank sum test[b]		Relative abundance				Prevalence			
				Philippines		AMP5[c]		Philippines		AMP[c]	
Genus	Species	*p*	*q*	BB	P	BB	P	BB	P	BB	P
Bacteroides		7.6E-07	9.7E-05	0.066	0.005	0.140	0.064	1.000	0.800	1.000	1.000
Bacteroides	*vulgatus***	3.7E-06	3.5E-04	0.045	0.004	0.095	0.039	1.000	0.720	0.995	1.000
Bacteroides	*fragilis*	6.4E-04	1.8E-02	0.011	0.001	0.006	0.008	0.722	0.240	0.652	0.447
Bacteroides	*uniformis*	2.1E-04	9.9E-03	0.004	0.000	0.014	0.005	0.722	0.200	0.857	0.753
Bacteroides	*distasonis*	1.3E-05	1.0E-03	0.005	0.000	0.006	0.003	0.833	0.240	0.843	0.812
Parabacteroides		5.1E-05	2.8E-03	0.007	0.001	0.010	0.006	0.944	0.560	0.914	0.953
Prevotella		3.7E-08	7.2E-06	0.009	0.226	0.044	0.212	0.500	1.000	0.410	0.847
Prevotella	*copri*	3.1E-08	7.2E-06	0.005	0.201	0.037	0.191	0.444	1.000	0.352	0.812
Prevotella	*stercorea*	7.8E-04	2.0E-02	0.004	0.017	0.005	0.015	0.167	0.720	0.157	0.529
Alloprevotella	*rava**	2.6E-04	1.0E-02	0.000	0.010	0.001	0.009	0.056	0.600	0.081	0.412
Faecalibacterium		1.8E-03	3.6E-02	0.059	0.028	0.033	0.028	1.000	1.000	0.990	1.000
Faecalibacterium	*prausnitzii*	1.8E-03	3.6E-02	0.059	0.028	0.033	0.028	1.000	1.000	0.990	1.000
Succinivibrio		5.5E-04	1.6E-02	0.000	0.041	0.000	0.004	0.056	0.560	0.019	0.200
Succinivibrio	*dextrinosolvens**	5.5E-04	1.6E-02	0.000	0.041	0.000	0.004	0.056	0.560	0.019	0.200
Olsenella	*profusa**	1.7E-03	3.6E-02	0.002	0.012	0.001	0.004	0.722	1.000	0.548	0.894

[a]Taxonomy was determined by the 'uclust' consensus-taxonomy assignment to the Greengenes reference sequence database (gg_13_5) except for ones marked by asterisk, which were not identified to a certain taxonomic group.

*Closest species chosen by the RDP SequenceMatch search followed by Seqmatch Q400 program (see Material & Methods for details).

**It contains OTU closely related to *Altipes putredinis* .

[b]Wilcoxon rank-sum test was performed to examine the significance of differences in the relative abundance of each taxonomic group between P- and BB-type samples by using R 2.13.1. Bacterial groups showing the q value lower than 0.05 are shown.

[b]Five countries (Japan, China, Taiwan, Thailand, and Indonesia) analyzed in the AMP Phase-I study.
